# Inflammatory parameters predict etiologic patterns but do not allow for individual prediction of etiology in patients with CAP – Results from the German competence network CAPNETZ

**DOI:** 10.1186/1465-9921-10-65

**Published:** 2009-07-12

**Authors:** Stefan Krüger, Santiago Ewig, Jana Papassotiriou, Jan Kunde, Reinhard Marre, Heike von Baum, Norbert Suttor, Tobias Welte, the CAPNETZ study group

**Affiliations:** 1 Medical Clinic I, University Clinic RWTH Aachen, Germany; 2 Thoraxzentrum Ruhrgebiet, Kliniken für Pneumologie und Infektiologie, Ev. Krankenhaus Herne und Augusta Kranken-Anstalt Bochum, Germany; 3 Research Department, Brahms AG, Hennigsdorf, Germany; 4 Department of Medical Microbiology and Hygiene, University Hospital Ulm, Germany; 5 Department of Internal Medicine, Charite- University Medicine, Berlin, Germany; 6 Infectious Diseases and Pulmonary Medicine, Charite- University Medicine, Berlin, Germany; 7 Department of Pneumology, Hannover Medical School, University Clinic Hannover, Germany

## Abstract

**Background:**

Aim of this study was to evaluate the correlation of inflammatory markers procalcitonin (PCT), C-reactive protein (CRP) and leukocyte count (WBC) with microbiological etiology of CAP.

**Methods:**

We enrolled 1337 patients (62 ± 18 y, 45% f) with proven CAP. Extensive microbiological workup was performed. In all patients PCT, CRP, WBC and CRB-65 score were determined. Patients were classified according to microbial diagnosis and CRB-65 score.

**Results:**

In patients with typical bacterial CAP, levels of PCT, CRP and WBC were significantly higher compared to CAP of atypical or viral etiology. There were no significant differences in PCT, CRP and WBC in patients with atypical or viral etiology of CAP. In contrast to CRP and WBC, PCT markedly increased with severity of CAP as measured by CRB-65 score (p < 0.0001). In ROC analysis for discrimination of patients with CRB-65 scores > 1, AUC for PCT was 0.69 (95% CI 0.66 to 0.71), which was higher compared to CRP and WBC (p < 0.0001). CRB-65, PCT, CRP and WBC were higher (p < 0.0001) in hospitalised patients in comparison to outpatients.

**Conclusion:**

PCT, CRP and WBC are highest in typical bacterial etiology in CAP but do not allow individual prediction of etiology. In contrast to CRP and WBC, PCT is useful in severity assessment of CAP.

## Introduction

Several inflammatory markers, e.g. C-reactive protein (CRP) and leukocyte count (WBC) are used in the diagnostics of pulmonary infections. However, they are unspecific and not helpful for the differentiation of bacterial or viral etiology of pneumonia [1-3]. Procalcitonin (PCT) is a promising alternative in this regard. It rapidly increases in bacterial infections but remains low in viral diseases. High plasma concentrations of PCT are typically seen in sepsis, meningitis and pneumonia [4-9]. PCT also seems to be a prognostic factor in sepsis or pneumonia [10-12].

Since June 2002 the German competence network CAPNETZ, funded by the Federal Ministry of Education and Research, samples data from inpatients and outpatients with CAP. CAPNETZ incorporates ten local clinical centres (LCC) spread over Germany. More information about CAPNETZ is available on the website [13]. This framework offers a good opportunity to study the value of inflammatory markers in the etiological diagnosis of CAP.

Thus, the aim of our study was to investigate whether inflammatory markers at admission are helpful to predict the microbiological etiology in CAP patients.

## Methods

### Patients

Within CAPNETZ all new CAP cases are reported via a network of sentinel practices and hospitals to the study monitor of the corresponding local clinical centre (LCC). The study monitor of the LCC includes the patient in CAPNETZ by applying the following criteria: age ≥ 18 years, a pulmonary infiltrate diagnosed by chest x-ray, clinical symptoms consisting of cough or purulent sputum or positive auscultation. Exclusion criteria are age < 18 years, acquired or therapeutically induced immune deficiency, florid tuberculosis or a possible nosocomial genesis of infection (hospitalisation less than four weeks prior to infection). After inclusion in CAPNETZ all clinical parameters of the patients are stored in an electronic database. CAP patients of ten LCC were included. Written informed consent was obtained from every patient prior to inclusion in the study. The study was approved by the local ethical committee. Our study was not an intervention study with implementation of standardized criteria for the diagnosis and therapy of CAP according to guidelines, biomarker levels or CRB-65 score.

### Microbiological diagnostics

At the point of inclusion into the study blood samples were taken for the determination of PCT, CRP and WBC, blood culture and serological testing for *Chlamydia pneumoniae *and *Mycoplasma pneumoniae*. Sputum samples and pharyngeal aspiration were taken from every patient whenever possible to test for bacteria and viruses according to standard procedures. Urine samples were collected and tested whenever possible for *Legionella pneumophila *and *Streptococcus pneumoniae *with an antigen test.

Sputum was Gram stained. Representative sputum originating from the lower respiratory tract was validated by the criteria > 25 granulocytes and < 10 epithelial cells per low power field (total magnification × 100). Validated sputum, blood culture samples and undiluted and serially diluted tracheobronchial aspirates were plated on the following media: blood-sheep agar, CDC agar, chocolate agar as well as Sabouraud agar. Urine was tested for the presence of *Streptococcus pneumoniae *and *Legionella spp*. antigen. Identification of microorganisms and susceptibility testing was performed according to standard methods.

Infectious etiology of pneumonia was classified as definite if one of the following criteria were met: 1) blood cultures yielding a bacterial or fungal pathogen (in the absence of an apparent extrapulmonary focus); 2) tracheobronchial secretions: at least ++ growth of one of the species defined as pathogens; 3) a valid sputum sample (leukocytes 25 per 10× field) yielded one or more predominant bacterial pathogens or 3+ growth. The following species were regarded as potential pathogens: *Streptococcus pneumoniae*, *Haemophilus influenzae*, *Klebsiella pneumoniae*, *Escherichia coli *and other enterobacterial species, *Pseudomonas aeruginosa*, *Moraxella catarrhalis*, *Stenotrophomonas maltophilia*; 4) *Chlamydia pneumoniae: *(IgM ≥ 1:32) and/or PCR positive in at least two different laboratories; 5) *Legionella*: bacterial growth in respiratory secretions or detection of urinary antigen or detection of legionella specific DNA by PCR; 6) *Mycoplasma pneumoniae: *PCR positive; 7) bacterial growth in cultures of TBAS ≥ 10^5 ^cfu/ml; 8) positive urinary antigen for *Streptococcus pneumoniae*; 9) PCR positive for Influenzavirus A and B, RS-Virus, Adenovirus, Enterovirus. 10) Aspergillus spp. were accepted as definite in the presence of concomitant lung abscess and/or histologic confirmation. Candida albicans was only accepted as the causative agent if it occurred in high numbers in purulent sputum (25 and more leukocytes per field).

### Determination of PCT, CRP and leukocyte count

Leukocyte count (WBC) was determined by the hospital laboratory. Serum CRP was measured by nephelometry with a commercially available assay (Behring Diagnostics, Marburg, Germany). Serum PCT was determined by an immunofluorescent assay (B.R.A.H.M.S PCT sensitive KRYPTOR, B.R.A.H.M.S AG, Henningsdorf, Germany). All serum samples for PCT testing were centrally stored at -70°C in the LCC Ulm until measurement. The assay requires 50 μl of serum, EDTA or heparin plasma, has a functional assay sensitivity (defined as lowest value with an interassay CV <20%) of 0.06 ng/mL and a lower detection limit of 0.02 ng/mL. Laboratory measurements were performed in a blinded fashion without knowledge of the microbiological results.

### Determination of CRB-65

The CRB-65 score consists of four variables: confusion, respiratory rate ≥ 30/min, systolic blood pressure < 90 mm Hg or diastolic blood pressure ≤ 60 mmHg, age ≥ 65 years [14,15]. One point is given for each parameter present which results in CRB-65 scores of 0–4. For each patient the CRB-65 score was calculated with patient data ascertained at first presentation.

### Statistics

We performed statistical analysis with Graph Pad Prism 4.0 and tested distribution with the Kolmogorov-Smirnov test. Two group comparisons of nonparametric data were performed by the Mann-Whitney U-test. For multigroup comparisons, Kruskal-Wallis one-way analysis of variance test was used. Frequency comparison was done using the χ^2^-test. We constructed Receiver-operating-characteristics (ROC) curves using MedCalc statistical software and determined the area under the curve (AUC). All statistical tests were 2-tailed and a p-value < 0.05 was considered statistically significant.

## Results

### Patients

In 1337 patients mean age was 62 ± 18 years (range 18 to 98 years), and 55% were male. The causative pathogen was identified in 472 patients (35.3%) [typical bacterial infection: n = 185, atypical bacterial infection n = 190, viral infection n = 39; mixed infections with two or more pathogens (n = 58) including the following combinations: two or more typical or atypical bacteria, typical bacterial plus atypical bacterial infection, typical or atypical bacterial infection plus viruses]. Organisms included in the definition of bacterial pneumonia and pneumonia caused by atypical pathogens and viruses are shown in table 1. 898 patients (67.2%) were hospitalised, 439 (32.8%) were outpatients.

**Table 1:** Causative microorganisms in CAP patients

**Etiology**	**Total**	**Inpatients**	**Outpatients**
Typical bacterial pathogens	185 (13.8%)	139 (10.4%)	46 (3.4.%)
*S. pneumoniae*	122 (9.1%)	95 (7.1%)	27 (2.0%)
*H. influenzae*	13 (1.0%)	8 (0.6%)	5(0.4%)
*Haemophilus spp*	2 (0.1%)	1 (0.1%)	1 (0.1%)
*E. coli*	9 (0.7%)	7 (0.5%)	2 (0.1%)
*S. aureus*	12 (0.9%)	8 (0.6%)	4 (0.3%)
*Moraxella catarrhalis*	5 (0.4%)	1 (0.1%)	4 (0.3%)
*Enterobacter spp*.	5 (0.4%)	5 (0.4%)	0
*Proteus mirabilis*	2 (0.1%)	2 (0.1%)	0
*Pseudomonas spp*.	1 (0.1%)	1 (0.1%)	0
*Pseudomonas aeruginosa*	1 (0.1%)	1 (0.1%)	0
*Citrobacter spp*	2 (0.1%)	0	2 (0.1%)
*Enterococcus faecium*	1 (0.1%)	1 (0.1%)	0
*Klebsiella pneumonite*	2 (0.1%)	2 (0.1%)	0
*Klebsiella oxytoca*	1 (0.1%)	1 (0.1%)	0
*Streptococcus agalactiae*	4 (0.3%)	3 (0.2%)	1 (0.1%)
*Streptococcus pyogenes*	1 (0.1%)	1 (0.1%)	0
*Streptococcus acidominimus*	1 (0.1%)	1 (0.1%)	0
Prevotella spp.	1 (0.1%)	1 (0.1%)	0

**Atypical pathogens**	190 (14.2%)	115 (8.6%)	75 (6.6%)
*M. pneumoniae*	140 (10.5%)	80 (6.0%)	60 (4.5%)
*Legionella spp*	48 (3.6%)	34 (2.5%)	14 (1.0%)
*Chl. pneumoniae*	2 (0.1%)	1 (0.1%)	1 (0.1%)

**Viruses**	39 (2.9%)	25 (1.9%)	14 (1.0%)
*Adenovirus*	2 (2.2%)	2 (0.1%)	14 (1.0)
*Influenza A*	33 (2.5%)	19 (1.4%)	0
*RS Virus*	3 (0.2%)	3 (0.2%)	0
Enterovirus	1 (0.1%)	1 (0.1%)	

**Mixed**	58 (4.3%)	42 (3.1%)	16 (1.2%)

**Unknown**	865 (64.7%)	577 (43.2%)	288 (21.5%)

**Total**	589 (100%)	384 (100%)	205 (100%)

Data are presented as number of cases (percentages of total number)

**Figure 1:**
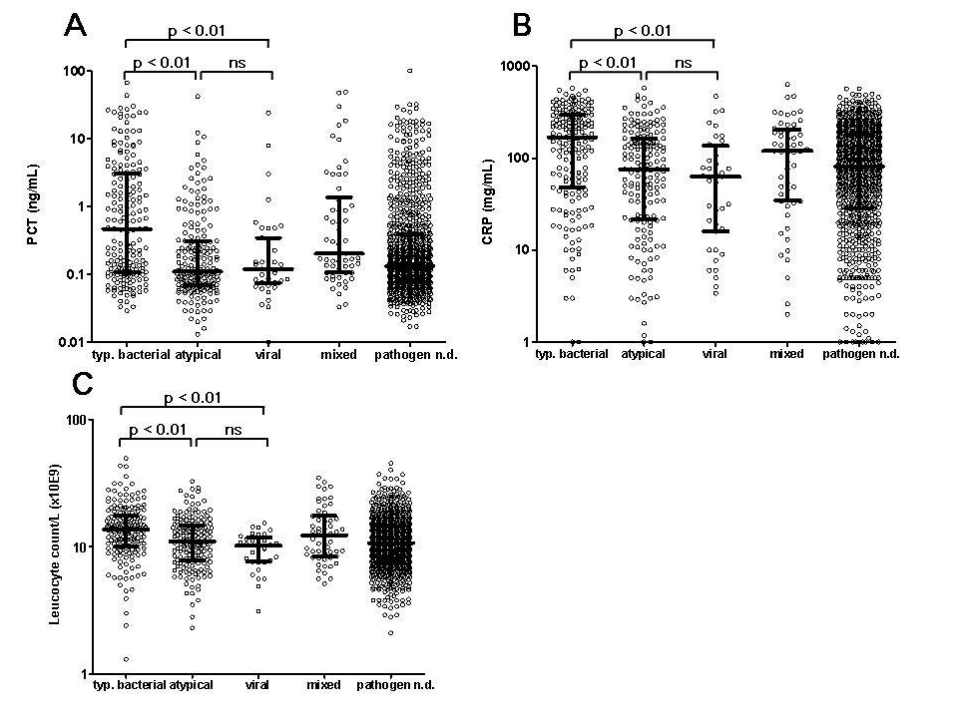
**Admission levels of PCT (a), CRP (b) and WBC (c) in CAP patients with classical bacterial, atypical, viral, "mixed" or unknown etiology**. The scatterplots represent all data. Median values with interquartile ranges are shown. ns = no significant difference; n.d. = unknown

The etiologic distribution of the causative pathogens is summarized in table 1. Patients with proven typical bacterial etiology showed significantly higher PCT levels (figure 1a), CRP levels (figure 1b) and WBC (figure 1c) compared to patients with atypical or viral etiology. A PCT cut-off level of 0.1 ng/mL showed an odds ratio of 8.3 (95% CI 4.8 to 14.5) and a cut-off level of 0.25 ng/mL an odds ratio of 3.2 (95% CI 2.1 to 5.0) to differentiate *S. pneumoniae *CAP from CAP due to atypical or viral etiology. Levels of PCT, CRP and WBC were comparable in patients with atypical or viral etiology.

### CRB-65 score

The severity of pneumonia was assessed using the CRB-65 score. Overall, the mean CRB-65 score was 0.96 ± 0.88 ranging from class 0 to 4 (class 0, n = 416; class 1, n = 497; class 2, n = 240, class 3, n = 46; class 4, n = 10). The distribution of the CRB-65 scores was comparable in patients with CAP due to typical bacterial etiology (mean CRB-65 score: 0.98 ± 0.92), atypical bacterial etiology (mean: 0.79 ± 0.84), viral (mean: 1.10 ± 0.85), "mixed" (mean: 0.98 ± 0.92) or unknown etiology (mean: 0.97 ± 0.87).

### Correlation of PCT, CRP and WBC to CRB-65 class

**Figure 2:**
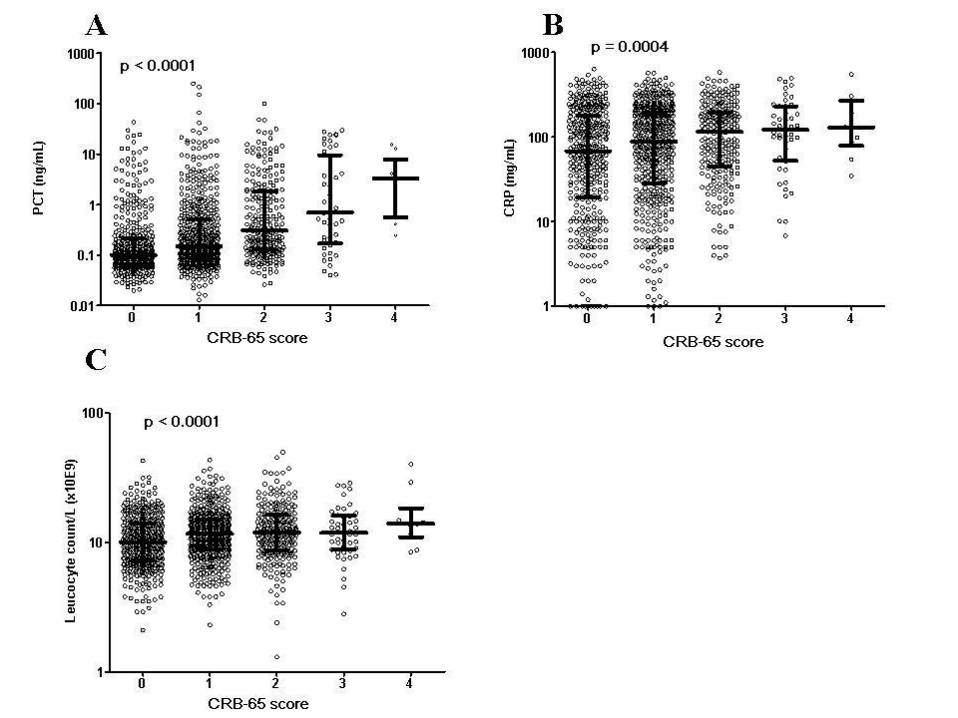
**Admission levels of PCT (a), CRP (b) and WBC (c) in CAP patients classified into CRB-65 classes 0–4**. The scatterplots represent all data. Median values with interquartile ranges are shown. Results of Kruskal-Wallis one-way analysis of variance are shown. ns = no significant difference.

Median levels of PCT, CRP and WBC were calculated according to CRB-65 severity class. PCT levels increased with increasing severity of CAP (p < 0.0001, figure 2a). Median PCT value was 0.10 ng/mL (range 0.002 to 43.31 ng/mL) in CRB-65 class 0; 0.15 ng/mL (range, 0.01 to 250.22 ng/mL) in class 1; 0.31 ng/mL (range, 0.03 to 100.00 ng/mL) in class 2; 0.70 ng/mL (range, 0.04 to 30.00 ng/mL) in class 3; and 3.33 ng/mL (range, 0.24 to 15.45 ng/mL) in class 4. Median PCT levels in patients with non-severe CAP (defined as CRB-65 class 0–1) were significantly lower (0.12 ng/mL) as compared to patients with severe CAP (defined as CRB-65 class 2–4; 0.36 ng/mL, p < 0.0001). In 410 patients PCT levels were < 0.1 ng/mL. These patients were classified into lower CRB-65 classes (mean 0.62 ± 0.72) compared to those patients with PCT levels ≥ 0.1 ng/mL (mean 1.13 ± 0.90, p < 0.0001). The distribution of CRP values is shown in figure 2b. Only a moderate increase with the severity of CAP could be observed. The CRB-65 classes 0 and 2 showed a significant difference in their CRP levels (p < 0.01), all other groups showed no significant difference. There was a significant difference (p = 0.0037) between median CRP values in patients classified into CRB-65 risk classes 0 and 1 (78.0 mg/L; 0.00 mg/mL – 634.00 mg/mL) and patients classified into CRB-65 classes 2–4 (118.0 mg/L; 0.50 mg/mL – 580.00 mg/mL)

Figure 2c shows the distribution of WBC in different CRB-65 classes. Median WBC in patients with non-severe CAP (defined as CRB-65 class 0–1) (10.9 G/L; 2.10 G/L – 43.20 G/L) was significantly (p = 0.0002) lower as compared to patients with severe CAP (defined as CRB-65 class 2–4; 12.05 G/L, 2.10 G/L – 49.60 G/L).

Receiver operating characteristic curves illustrate the accuracy of PCT, CRP and WBC to predict severe CAP (defined as CRB-65 > 1). The AUC for PCT was 0.69 (95% CI 0.66 to 0.71) demonstrating fair to good discriminatory power. At a cut-off level of 0.1 ng/mL an odds ratio of 3.7 (95% CI 2.6 to 5.2) was calculated for the prediction of severe CAP. The AUCs for CRP (0.57, 95% CI 0.54 to 0.60) and WBC (0.56, 95% CI 0.53 to 0.590) were significantly lower (p < 0.0001) and demonstrated poor discriminatory power.

### Outpatients and hospitalised CAP patients

Outpatients were classified into lower CRB-65 classes (mean 0.43 ± 0.57) compared to hospitalised patients (mean 1.20 ± 0.89, p < 0.0001). Median PCT, CRP, and WBC values were significantly higher in hospitalised patients compared to outpatients. In ROC curve analysis where sensitivity was calculated among those patients who were hospitalised and specificity was assessed among patients who were treated as outpatients the AUC for CRB-65 was 0.74 (95% CI 0.72 to 0.77) demonstrating good discriminatory power. For PCT, AUC was significantly higher (0.79, 95% CI 0.76 to 0.81, p = 0.02). At a cut-off level of 0.1 ng/mL an odds ratio of 6.8 (95% CI 5.3 to 8.8) was calculated for the prediction of hospitalisation. The AUCs for CRP (0.73, 95% CI 0.71 to 0.76, p < 0.001) and WBC (0.70, 95% CI 0.68 to 0.73, p < 0.001) were significantly lower compared to PCT.

### Atypical pathogens

**Figure 3:**
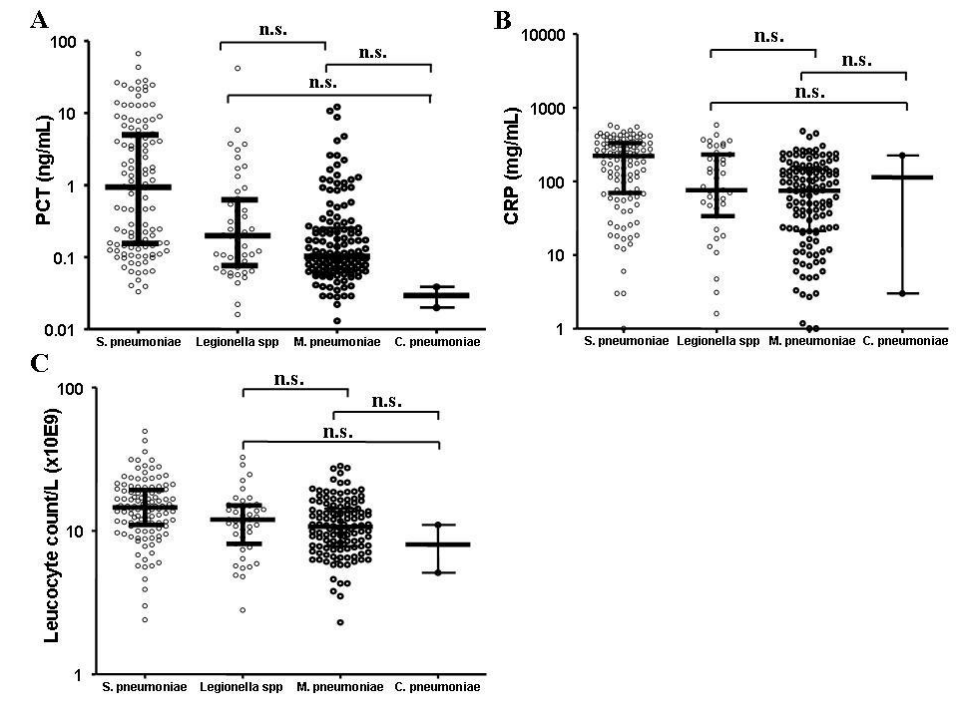
**Admission levels of PCT (a), CRP (b) and WBC (c) in CAP patients with pneumococcal and atypical etiology of CAP**. The scatterplots represent all data. Median values with interquartile ranges are shown. ns = no significant difference.

In the study population we identified 48 patients with *Legionella pneumophila*, 140 with *Mycoplasma pneumoniae *and only two patients with *Chlamydia pneumoniae*. In patients with CAP caused by *Legionella pneumophila*, *Mycoplasma pneumoniae *or *Chlamydia pneumoniae*, (Figure 3) there were no significant differences in PCT (0.20 ng/mL; 0.02 ng/mL – 41.77 ng/mL vs. 0.10 ng/mL; 0.01 ng/mL – 12.14 ng/mL vs. 0.03 ng/mL; 0.02 ng/mL – 0.04 ng/mL, n.s.), CRP (76.00 mg/mL; 0.50 mg/mL – 580.00 mg/mL vs. 74.75 mg/mL; 0.80 mg/mL – 480.00 mg/mL vs. 113.5 mg/mL; 3.00 mg/mL – 224.00 mg/mL, n.s.) and WBC (12.00 G/L; 2.80 G/L – 32.60 G/L vs. 10.70 G/L; 2.3 G/L – 28.40 G/L vs. 8.05 G/L; 5.1 G/L – 11.00 G/L, n.s.).

## Discussion

The current study demonstrated that a) PCT, CRP and WBC values are significantly higher in CAP due to classical bacterial pathogens compared to atypical bacterial or viral pneumonia, b) PCT levels increase with an increasing CRB-65 score as a marker of the severity of CAP and c) PCT levels and CRB-65 score show a comparable power to detect CAP patients that will be hospitalised, whereas CRP or WBC have very poor discriminatory power in this point.

In CAP it is essential to assess disease severity to optimise therapeutic decisions, e.g. about hospitalisation, ICU admission and choice of antibiotic treatment [14-18]. Different scoring systems have been developed for a more objective assessment of CAP severity. Based on the modified severity assessment score of the British Thoracic Society [14] the simple CURB score was developed [15]. In a primary care setting blood urea results are not directly available. Therefore the CURB score has been modified to the CRB-65 score that includes only clinical variables. Blood urea is excluded and instead age ≥ 65 years is used as a variable. Risk assessment by the CRB-65 score yielded in results equal to the CURB score in CAP patients [19]. CAP patients with a CRB-65 score of 0–1 have a very low mortality risk and can be treated as outpatients [20]. Patients with a CRB-65 score of ≥ 2 are at intermediate (score 2) or high risk (score 3–4) and should be treated in hospital. In our study, CRB-65 score was significantly lower in outpatients but was not influenced by the microbiological etiology of CAP. In a former study of our group we could demonstrate that PCT levels on admission predict the severity and outcome of CAP with a similar prognostic accuracy as the CRB-65 score and a higher prognostic accuracy compared to WBC and CRP [12].

To our knowledge, this is the largest study to date performed in adults with CAP in which PCT and CRP serum levels and WBC have been evaluated in all patients. A detailed analysis of the correlation of these inflammatory markers with CRB-65 score has not been performed in a larger CAP population before. In a recently published study, Masia et al. found that PCT contribution to the evaluation of patients with CAP varies according to severity of pneumonia [11]. The authors described no differences in PCT levels between major etiologic groups when the whole sample of patients with CAP was considered. However, when patients were stratified according to PSI score, the highest PCT levels tended to predict bacterial etiology in patients with a low PSI score (risk classes I and II). No differences in PCT levels were found between major etiologic groups in patients with higher PSI scores (risk classes III-V). CRP values were not reported in this study. In contrast to the study by Masia et al. we found significantly higher PCT levels in classical bacterial CAP compared to atypical bacterial or viral pneumonia. This might be explained by the higher number of patients that were evaluated in our study (1337 vs. 185 patients), so that the sample size in the different etiologic groups was higher, which resulted in a higher statistical power.

Our study confirms the findings of previous studies that PCT is a good predictor of severity of pneumonia [8,10,11,21]. Patients with a higher CRB-65 score had significantly higher PCT levels. The discriminatory power of the CRB-65 score and PCT for the prediction of hospitalisation of CAP patients was comparable. A PCT level > 0.1 ng/mL constitutes a 3.7-fold higher risk to suffer from severe CAP (defined as CRB-65 score >1) and a 6.8-fold higher risk to be hospitalised. CRP and WBC were not helpful for the discrimination of low risk and high risk patients and the prediction of hospitalisation.

CAP can have various etiologies. In our study, classical and atypical bacteria were the most frequent pathogens in CAP. The distribution of pathogens in our study was comparable to a previous study including patients with severe CAP [22]. We found significantly higher PCT, and CRP concentrations as well as WBC in patients with classical bacterial compared to atypical bacterial CAP. In contrast to CRP and WBC, PCT may have one very important potential – it might be a marker for clinically relevant infections and could be used to decide if antibiotic treatment should be initiated or not [23,24]. Antibiotic use might be discouraged in patients with low PCT levels (e.g. < 0.1 ng/mL). In a randomised controlled study with patients with lower respiratory tract infections, the use of antibiotics could be reduced by PCT guided therapy by 50% without any negative impact on clinical outcome [23]. In a second randomised trial, Christ-Crain et al. studied PCT guided antibiotic therapy in patients with CAP [24]. Antibiotic therapy was discouraged if PCT was < 0.25 ng/mL. As a result, PCT guided therapy significantly reduced total antibiotic exposure, antibiotic prescription on admission and antibiotic treatment duration compared to treatment according to current guidelines. The important effect of this study on clinical management of CAP patients, treatment costs and development of microbiological resistance has to be taken into account. The results of the study by Christ-Crain et al. can be partially explained by our findings. We could demonstrate that CAP patients with lower severity of disease had significantly lower PCT levels. In conclusion, patients with lower severity of disease might probably need less intensive antibiotic therapy. A PCT cut-off level of 0.1 ng/mL showed an odds ratio of 8.3 to differentiate *S. pneumoniae *CAP from CAP due to atypical or viral etiology. However, it was not possible to definitively differentiate between CAP due to *S. pneumoniae *or other etiologies, so that a single PCT measurement at admission seems not allow the decision to prescribe a small or broad spectrum antibiotic. CAP with viral or atypical etiology showed comparable levels of inflammatory biomarkers, so that a differentiation of both etiologies and the consecutive choice to start antibiotic therapy was not possible in the individual patient. Furthermore, there is the dilemma that higher PCT levels are indicative of *S. pneumoniae *etiology on the one hand but also of more severe CAP on the other hand.

PCT levels in our study are lower than those reported in other studies including patients with lower respiratory tract infections [23] or CAP [10,25]. This could be explained by the fact, that these previous studies included hospitalised patients in contrast to our study with a percentage of 32.8% outpatients. Although approximately 80% of CAP patients are not hospitalised, data for CAP outpatients is very rare. Thus, one important goal of the CAPNETZ study is to collect representative data about outpatients. The severity of disease in outpatients is usually lower, which could be demonstrated by a lower CRB-65 score in this group. As a consequence, these less severe ill CAP patients show lower values of PCT. In other studies that also included CAP outpatients, PCT levels were lower and comparable with our results [8,26].

CRP is an early sensitive but non-specific marker of inflammation. Long ago, CRP was initially discovered as a test for patients with pneumococcal pneumonia [27]. Interestingly, there is only limited data on CRP in larger studies including CAP patients. In one small study with 28 patients it could be demonstrated that serial CRP measurements are helpful for the prediction of antibiotic treatment failure or the development of infective complications [28]. Patients with high CRP levels show a longer duration of fever, longer hospital stay, and recover less often after discharge, but CRP is not associated with a higher mortality [29]. One recent study suggested higher CRP levels in *Legionella pneumophila *infection but found no correlation of CRP to the severity of the disease as measured by the PSI score [30]. This corresponds with our results showing no increase in CRP concentrations with the severity of CAP as measured by the CRB-65 score. In pediatric patients, serum CRP was not useful to distinguish between classical bacterial, atypical or viral pneumonia [2,31]. In contrast, Almirall et al. found in 201 patients with CAP higher levels of CRP in case of an infection by *S. pneumoniae *and *Legionella pneumophila *compared to other infectious agents [32]. Almirall et al. also described significantly lower CRP values in outpatients than hospitalised patients. In our study group, CRP levels of outpatients were lower compared to inpatients, too. The same holds true for PCT and WBC, which both were significantly lower in outpatients in our study.

The present study has some limitations. Despite intensive microbiological diagnostics, the etiology remained unknown in 64.7%. This low sensitivity of microbiological tests is well known from other pneumonia studies. The etiology of CAP has been studied in various patient populations, regions, settings and with different diagnostic methodologies. A constant finding is the failure to detect a pathogen in up to 60% of cases of hospitalized patients with CAP [14,33,34]. There are several factors that may reduce the diagnostic yield in our study as well as in the previous ones. First, ambulatory antimicrobial pre-treatment is very important. Nearly one third of patients are pre-treated with antibiotics on hospital admission. Fang et al. clearly showed the decline in diagnostic yield in the presence of antibiotic pre-treatment [35]. Many cases of unknown etiology may be caused by *Streptococcus pneumoniae*, a pathogen which is easily missed after one single dose of antimicrobial treatment [36]. A second factor might be that *Mycoplasma pneumoniae *and *Chlamydia pneumoniae *might be often not recognised due to diagnostic problems, but represent important causes of CAP. Third, another point that could explain the low number of patients with a microbiological diagnosis is the fact that 32.8% of our patients included were outpatients. Outpatients are usually less severely ill. The percentage of outpatients that present with representative sputum or bacteraemia that increase diagnostic yield is lower compared to patients that are hospitalized. An important confounder which may have accounted for a large part of the undiagnosed cases is incomplete diagnostic work-up, especially in outpatients. A more extensive and aggressive diagnostic approach may have increased the diagnostic yield. However, even when using a most comprehensive diagnostic approach the diagnostic yield is at maximum 70–80% [14,33]. The CAPNETZ study is a huge population based study that includes outpatients and inpatients. The application of more invasive procedures such as bronchoscopy including bronchial washing and brushing is not feasible and realistic in such a study and was therefore omitted. Interestingly, it was previously shown that mortality is not different between patients with and without known etiology of CAP [37].

In conclusion, appropriate tools for establishing microbial diagnosis and assessing severity of disease in CAP would be helpful for optimal management of this disease. Measurement of PCT, CRP, and WBC may be useful to predict typical bacterial pneumonia, since elevated levels were observed in comparison to atypical bacterial and viral pneumonia. However, the inflammatory markers do not allow an individual prediction of microbial etiology of CAP. PCT might be a valuable tool helping clinicians – in combination with scoring systems- to identify clinically relevant infections, to assess a patient's risk profile, and to improve therapeutic decision making as well as decisions about hospitalisation and ICU admission.

## Competing interests

JP and JK are employees of BRAHMS AG, the manufacturer of the assay B.R.A.H.M.S PCT sensitive KRYPTOR, B.R.A.H.M.S AG, Henningsdorf, Germany. JP and JK do not own stock or options in the company. TW received funds for speaking at symposia organized on behalf of BRAHMS AG. All other authors: none to declare.

## Authors' contributions

SK helped planning the study, performed data processing and interpretation and wrote the manuscript. NS, RM and TW organized CAPNETZ and data processing, planned the study and helped with data interpretation and with the manuscript. SE helped with data interpretation and with the manuscript. JP and JK helped planning the study, performed data processing and interpretation and helped with the manuscript. HvB organised microbiological work in the central study unit.

## References

[B1] Korppi M, Heiskanen-Kosma T, Leinonen M (1997). White blood cells, C-reactive protein, and erythrocyte sedimentation rate in pneumococcal pneumonia in children. Eur Respir J.

[B2] Heiskanen-Kosma T, Korppi M (2000). Serum C-reactive protein cannot differentiate bacterial and viral aetiology of community-acquired pneumonia in children in primary healthcare settings. Scand J Infect Dis.

[B3] Clyne B, Olshaker JS (1999). The C-reactive protein. J Emerg Med.

[B4] Boussekey N, Leroy O, Alfandari S, Devos P, Georges H, Guery B (2006). Procalcitonin kinetics in the prognosis of severe community-acquired pneumonia. Intensive Care Med.

[B5] Boussekey N, Leroy O, Georges H, Devos P, d'Escrivan T, Guery B (2005). Diagnostic and prognostic values of admission procalcitonin levels in community-acquired pneumonia in an intensive care unit. Infection.

[B6] Brunkhorst FM, Al-Nawas B, Krummenauer F, Forycki ZF, Shah PM (2002). Procalcitonin, C-reactive protein and APACHE II score for risk evaluation in patients with severe pneumonia. Clin Microbiol Infect.

[B7] Harbarth S, Holeckova K, Froidevaux C, Pittet D, Ricou B, Grau GE, Vadas L, Pugin J, Geneva Sepsis Network (2001). Diagnostic value of procalcitonin, interleukin-6, and interleukin-8 in critically ill patients admitted with suspected sepsis. Am J Respir Crit Care Med.

[B8] Hausfater P, Garric S, Ayed SB, Rosenheim M, Bernard M, Riou B (2002). Usefulness of procalcitonin as a marker of systemic infection in emergency department patients: a prospective study. Clin Infect Dis.

[B9] Oppert M, Reinicke A, Muller C, Barckow D, Frei U, Eckardt KU (2002). Elevations in procalcitonin but not C-reactive protein are associated with pneumonia after cardiopulmonary resuscitation. Resuscitation.

[B10] Hedlund J, Hansson LO (2000). Procalcitonin and C-reactive protein levels in community-acquired pneumonia: correlation with etiology and prognosis. Infection.

[B11] Masia M, Gutierrez F, Shum C, Padilla S, Navarro JC, Flores E, Hernandez I (2005). Usefulness of procalcitonin levels in community-acquired pneumonia according to the patients outcome research team pneumonia severity index. Chest.

[B12] Krüger S, Ewig S, Marre R, Papassotiriou J, Richter K, von Baum H, Suttorp N, Welte T (2008). Procalcitonin predicts patients at low risk of death from community- acquired pneumonia. Eur Resp J.

[B13] Welte T, Suttorp N, Marre R (2004). CAPNETZ-community-acquired pneumonia competence network. Infection.

[B14] Neill AM, Martin IR, Weir R, Anderson R, Chereshsky A, Epton MJ, Jackson R, Schousboe M, Frampton C, Hutton S, Chambers ST, Town GI (1996). Community acquired pneumonia: aetiology and usefulness of severity criteria on admission. Thorax.

[B15] Lim WS, Eerden MM van der, Laing R, Boersma WG, Karalus N, Town GI, Lewis SA, Macfarlane JT (2003). Defining community acquired pneumonia severity on presentation to hospital: an international derivation and validation study. Thorax.

[B16] Fine MJ, Auble TE, Yealy DM, Hanusa BH, Weissfeld LA, Singer DE, Coley CM, Marrie TJ, Kapoor WN (1997). A prediction rule to identify low-risk patients with community-acquired pneumonia. N Engl J Med.

[B17] Buising KL, Thursky KA, Black JF, MacGregor L, Street AC, Kennedy MP, Brown GV (2006). A prospective comparison of severity scores for community acquired pneumonia: reconsidering what is meant by severe pneumonia. Thorax.

[B18] Niederman MS, Mandell LA, Anzueto A, Bass JB, Broughton WA, Campbell GD, Dean N, File T, Fine MJ, Gross PA, Martinez F, Marrie TJ, Plouffe JF, Ramirez J, Sarosi GA, Torres A, Wilson R, Yu VL, American Thoracic Society (2001). Guidelines for the management of adults with community-acquired pneumonia. Diagnosis, assessment of severity, antimicrobial therapy, and prevention. Am J Respir Crit Care Med.

[B19] Bauer TT, Ewig S, Marre R, Suttorp N, Welte T, The Capnetz Study Group (2006). CRB-65 predicts death from community-acquired pneumonia. J Intern Med.

[B20] Niederman MS, Feldmann C, Richards GA (2006). Combining information from prognostic scoring tools for CAP: an American view on how to get the best of all worlds. Eur Respir J.

[B21] Polzin A, Pletz M, Erbes R, Raffenberg M, Mauch H, Wagner S, Arndt G, Lode H (2003). Procalcitonin as a diagnostic tool in lower respiratory tract infections and tuberculosis. Eur Respir J.

[B22] Oosterheert JJ, Bonten MJ, Hak E, Schneider MM, Hoepelman AI (2003). Severe community-acquired pneumonia: what's in a name?. Curr Opin Infect Dis.

[B23] Christ-Crain M, Jaccard-Stolz D, Bingisser R, Gencay MM, Huber PR, Tamm M, Muller B (2004). Effect of procalcitonin-guided treatment on antibiotic use and outcome in lower respiratory tract infections: cluster-randomised, single-blinded intervention trial. Lancet.

[B24] Christ-Crain M, Stolz D, Bingisser R, Muller C, Miedinger D, Huber PR, Zimmerli W, Harbarth S, Tamm M, Muller B (2006). Procalcitonin-Guidance of Antibiotic Therapy in Community-Acquired Pneumonia – A Randomized Trial. Am J Respir Crit Care Med.

[B25] Toikka P, Irjala K, Juvén T, Virkki R, Mertsola J, Leinonen M, Ruuskanen O (2000). Serum procalcitonin, C-reactive protein and interleukin-6 for distinguishing bacterial and viral pneumonia in children. Pediatr Infect Dis J.

[B26] Korppi M, Remes S, Heiskanen-Kosma T (2003). Serum procalcitonin concentrations in bacterial pneumonia in children: a negative result in primary healthcare settings. Pediatr Pulmonol.

[B27] Tillett WS, Francis T (1930). Serological reactions in pneumonia with non-protein somatic fraction of pneumococcus. J Exp Med.

[B28] Smith RP, Lipworth BJ, Cree IA, Spiers EM, Winter JH (1995). C-reactive protein. A clinical marker in community-acquired pneumonia. Chest.

[B29] Ortqvist A, Hedlund J, Wretlind B, Carlstrom A, Kalin M (1995). Diagnostic and prognostic value of interleukin-6 and C-reactive protein in community-acquired pneumonia. Scand J Infect Dis.

[B30] Garcia Vazquez E, Martinez JA, Mensa J, Sanchez F, Marcos MA, de Roux A, Torres A (2003). C-reactive protein levels in community-acquired pneumonia. Eur Respir J.

[B31] Moulin F, Raymond J, Lorrot M, Marc E, Coste J, Iniguez JL, Kalifa G, Bohuon C, Gendrel D (2001). Procalcitonin in children admitted to hospital with community acquired pneumonia. Arch Dis Child.

[B32] Almirall J, Bolíbar I, Toran P, Pera G, Boquet X, Balanzó X, Sauca G, Community-Acquired Pneumonia Maresme Study Group (2004). Contribution of C-reactive protein to the diagnosis and assessment of severity of community-acquired pneumonia. Chest.

[B33] Ortqvist A, Hedlund J, Grillner L, Jalonen E, Kallings I, Leinonen M, Kalin M (1990). Aetiology, outcome and prognostic factors in patients with community-acquired pneumonia requiring hospitalization. Eur Respir J.

[B34] Bohte R, van Furth R, Broek PJ van den (1995). Aetiology of community-acquired pneumonia: a prospective study among adults requiring admission to hospital. Thorax.

[B35] Fang GD, Fine M, Orloff J, Arisumi D, Yu VL, Kapoor W, Grayston JT, Wang SP, Kohler R, Muder RR (1990). New and emerging etiologies for community-acquired pneumonia with implications for therapy. A prospective multicenter study of 359 cases. Medicine.

[B36] Farr BM, Kaiser DL, Harrison BDW, Connolly C (1989). Prediction of microbial aetiology at admission to hospital for pneumonia from the presenting clinical features. Thorax.

[B37] Ewig S, Torres A, Angeles Marcos M, Angrill J, Rañó A, de Roux A, Mensa J, Martínez JA, de la Bellacasa JP, Bauer T (2002). Factors associated with unknown aetiology in patients with community-acquired pneumonia. Eur Respir J.

